# Routine imaging guided by a 31-gene expression profile assay results in earlier detection of melanoma with decreased metastatic tumor burden compared to patients without surveillance imaging studies

**DOI:** 10.1007/s00403-023-02613-6

**Published:** 2023-03-28

**Authors:** Soneet Dhillon, Daniela Duarte-Bateman, Graham Fowler, Michael Norman Eun Hagstrom, Nathaniel Lampley, Shantel Olivares, Mónica Stella Fumero-Velázquez, Kathryn Vu, Jeffrey D. Wayne, Brian R. Gastman, John Vetto, Pedram Gerami

**Affiliations:** 1 Department of Dermatology, Feinberg School of Medicine, Northwestern University, 676 N. St. Clair Street, Suite 1765, Chicago, IL 60611 USA; 2 Department of Plastic Surgery, Cleveland Clinic Lerner Research Institute, Cleveland, OH USA; 3 Division of Surgical Oncology, Knight Cancer Institute, Oregon Health and Science University, Beaverton, USA; 4 Division of Surgical Oncology, Northwestern University, Chicago, IL USA; 5 Robert H. Lurie Cancer Center, Feinberg School of Medicine, Northwestern University, Chicago, IL USA

**Keywords:** Cutaneous melanoma, Gene expression profile, Metastasis, Surveillance imaging, Recurrence

## Abstract

Patients with early-stage disease typically have a good prognosis, but still have a risk of recurrence, even with negative sentinel lymph node biopsy (SLNB). This study explores the utility of routine imaging to detect metastases in patients with negative SLNB but high-risk 31 gene expression profile (31-GEP) scores. We retrospectively identified melanoma patients with negative SLNBs. Patients with high-risk GEP results were placed in the experimental group and patients without GEP testing were placed in the control group. Among both cohorts, recurrent melanoma groups were identified. The tumor burden at the time of recurrence and the time to recurrence were compared between experimental group patients with routine imaging and control group patients without imaging schedules. We identified 327 control patients and 307 experimental patients, of which 14.1% versus 20.5% had melanoma recurrence, respectively. Of the patients with recurrent melanoma, those in the experimental group were older (65.75 versus 59.20), had higher Breslow depths (3.72 mm versus 3.31 mm), and had advanced tumor staging (89.5% versus 71.4% of patients presenting clinical stage ≥ II) compared to the control group at primary diagnosis. However, melanoma recurrence was detected earlier (25.50 months versus 35.35 months) in the experimental group at a lower overall tumor burden (73.10 mm versus 27.60 mm). A higher percentage of experimental patients started immunotherapy when offered (76.3% and 67.9%). Patients who received routine imaging after high-risk GEP test scores had an earlier recurrence diagnosis with lower tumor burden, leading to better clinical outcomes.

## Introduction

Immunotherapies such as anti-programmed cell death ligand 1(anti-PD-1) immune checkpoint inhibitor (ICI) and anti-cytotoxic T lymphocyte antigen-2 (anti-CTLA-4) ICI, as well as targeted antitumor treatments, including *B-Raf* proto-oncogene (BRAF) and mitogen-activated protein-kinase-kinase (MEK) inhibitors, have revolutionized melanoma treatment [1, 2]. Follow-up data support the effectiveness of these newer therapies in improving progression-free survival (PFS) and overall survival (OS) [1–3]. Importantly, many trials involving these novel agents suggest greater efficacy when administered to patients with an initial lower tumor burden [4–8].

Routine imaging can effectively detect early relapse when there is a lower tumor burden [9–11]. For patients with stage IIB disease or greater, the National Comprehensive Cancer Network^®^ (NCCN^®^) recommends chest radiography, CT (CT), brain magnetic resonance imaging (MRI), and positron emission tomography and CT (PET/CT) every 3–12 months for 3 years at the discretion of the physician [12]. However, routine imaging is not a standard protocol for clinical stage 1 and 2 patients, and the guidelines for surveillance remain controversial.

Notably, recurrence in patients with early-stage disease is well documented and may be as high as 40–69% [12–14]. Additionally, AJCC and SEER data show that, excluding stage IV patients, 60% of patients who ultimately die from metastatic melanoma are stage 1 or 2 at the time of initial diagnosis [15–17]. Hence, a significant number of patients diagnosed with early-stage disease may have aggressive melanomas that may recur and ultimately result in death. Any method of determining which patients may most benefit from routine imaging should aim at identifying patients at a point of low total tumor burden, as they may have the greatest chance for cure or improved survival with immunotherapy or targeted therapy.

A 31-gene expression profile (31-GEP) test was introduced in 2013 and yields a continuous probability score between zero and one that stratifies the risk of melanoma disease recurrence. The score is assigned to four categories: low risk of recurrence (Class 1A; 0–0.41 and Class 1B; 0.42–0.49), and high risk of recurrence (Class 2A; 0.50–0.58 and Class 2B; 0.59–1) [18–21]. The 31-GEP Class has been demonstrated to be an independent predictor of recurrence, including nodal recurrence and distant metastasis-free survival (DMFS) in meta-analyses and multiple prospective and retrospective studies [20, 22]. This study looked at patients with a negative sentinel lymph node (SLN) biopsy and a high-risk 31-GEP result. We then selected patients with recurrence and compared the tumor burden between patients who underwent a routine imaging protocol to those who did not. Specifically, we compared our experimental cohort with a control cohort of patients with negative SLN biopsy results who did not have GEP testing and only had imaging studies as indicated by clinical symptoms to validate the utility of GEP results in guiding radiological surveillance. Patients with high-risk recurrence scores from GEP testing who were subsequently placed on routine imaging had an earlier recurrence diagnosis with lower tumor burden with a trend towards improved overall survival.

## Methods

### Study design and patient selection

**Fig. 1:**
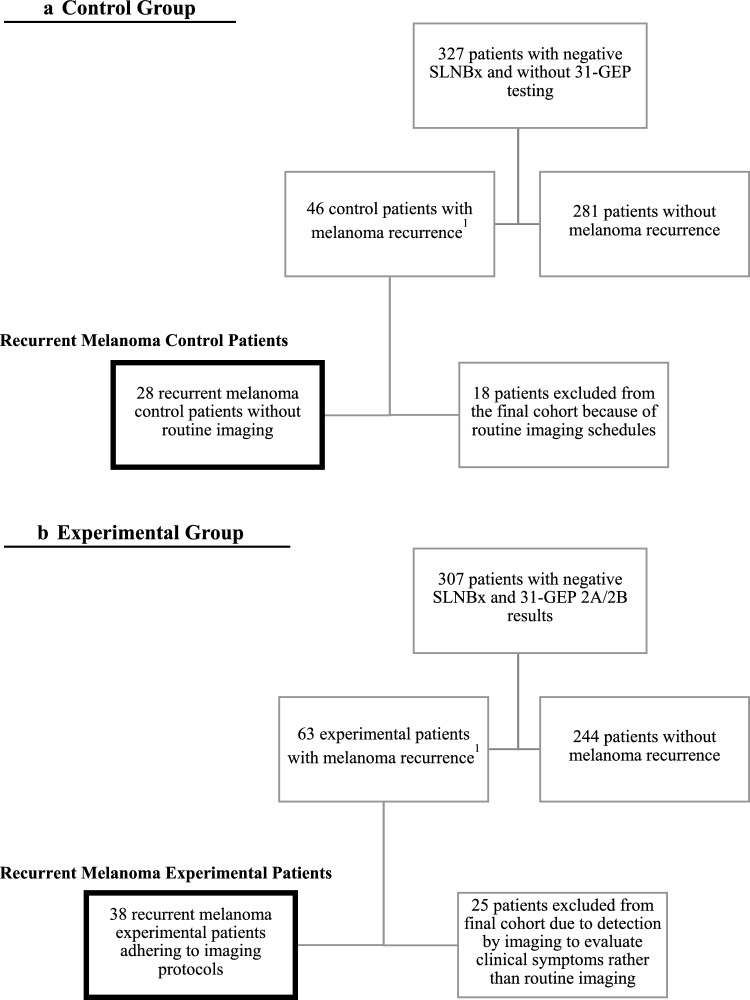
Flow diagram illustrating study participant eligibility and group allocation

Retrospective chart reviews were performed at Northwestern University, Cleveland Clinic, and Oregon Health & Science University. All patients with a confirmed melanoma diagnosis and a negative sentinel lymph node biopsy (pathologic Stage 1 or 2 disease at the initial diagnosis) were selected for this study (Fig. 1). Patients tested with a 31-GEP (Castle Biosciences, Friendswood, TX) and with a GEP Class result of 2A or 2B were recruited into the experimental group, while Class 1A and 1B patients were excluded from the study. Patients who were Class 2A/B but without a SLNB were excluded from this study. All patients without GEP testing were placed in the control group if scheduled routine imaging was not part of the follow-up plan. In this control group, imaging studies were driven by symptoms or physical exam findings. Within each group, subgroups were identified as patients with visceral or lymphatic melanoma recurrence versus those without recurrence. Patients with routine imaging in the melanoma recurrent control group or those who did not adhere to imaging schedules in the melanoma recurrent experimental group were excluded from the melanoma recurrence groups.

Tumor burden at the first date of detection of recurrence was compared between the experimental subgroup with recurrence versus the control subgroup with recurrence. Patients were only included in the experimental subgroup of recurrent disease patients if they adhered to routine imaging schedules and had metastasis detection because of routine imaging rather than imaging performed as a result of a clinical symptom. The first endpoint was the date of detection of the first evidence of recurrence and the date of the last chart review was the secondary endpoint. The primary outcome of this study was the total tumor burden calculated at the first identified time of any evident recurrence of melanoma. Although there were differences between sites and patients, the routine imaging protocol typically consisted of chest CT without contrast, abdominal pelvic CT with contrast, and brain MRIs with and without contrast at an average of 6-month intervals.

### Tumor burden measurement, treatment outcomes, and survival analysis

Imaging reports interpreted by attending radiologists were used to determine tumor burden. The first radiology report in which the diagnosis of metastatic melanoma was suspicious enough to provide a measurement of the tumor was used to determine the initial tumor burden and the date of the first recurrence. Hence, the first sign of measurable tumor burden was used as the primary endpoint. If multiple foci were identified, the measurements were added together to determine the total tumor burden. Additional foci identified in subsequent studies that were not present in the first imaging study were not included in the measurement. All imaging studies that were part of the initial workup identifying measurable tumor burden were included. The tumor burden measurement was calculated using an adapted version of the response evaluation criteria in solid tumors (RECIST) 1.1 criteria by calculating the unidimensional sum of all reported metastatic foci present in initial imaging studies (method of Dall’Olio et al. [23, 24]. Specifically, the measured sums of the longest single length of all included metastatic foci led to the total tumor burden. Unlike the RECIST criteria, which evaluate the change in tumor burden, no tumor exclusions were made based on the minimum tumor size or the maximum number of tumors present in any organ, and ultrasound (US) examination measurements were included to determine the initial metastatic foci present that were ultimately included in tumor burden measurement [24].

Patient charts were reviewed to determine if they were treated with immunotherapy or other agents after the first detection of melanoma recurrence. Patient survival data was determined using the second study endpoint, which was the date of the last review of the chart.

### Analysis

The tumor burden differences between the control and experimental groups were evaluated using a two-sample *t-*test with unequal variances, and a two-sided *P* value < 0.05 was considered statistically significant. The time to progression was from the diagnosis of primary melanoma to detection of visceral or lymphatic metastases. Kaplan–Meier estimates with the log-rank test analysis were used to assess months melanoma recurrence between the experimental and control groups. Descriptive statistics included sex, age, average Breslow depth, tumor staging, and the site of initial melanoma and recurrence. The Chi-Square and Kruskal–Wallis statistic were used to determine the significance between both groups among each descriptive variable. P values ≤ 0.05 were considered significant. Statistical analysis was performed using Microsoft Excel and XLSTAT 2022.

## Results

A total of 307 patients with stage 1 or 2 clinical disease and a GEP Class 2A/B result were included in the experimental group. In comparison, 327 stage 1 or 2 patients without GEP testing were included in the control group. There were 63 recurrences in the experimental group versus 46 in the control group, which was statistically significant (20.5% versus 14.1%, p-value 0.031). Among the 63 recurrences in the experimental group, 38 patients followed a routine imaging protocol, while 25 did not, so they were excluded from the primary endpoint analysis for tumor burden. None of the patients in the control group followed an imaging protocol.

**Fig. 2:**
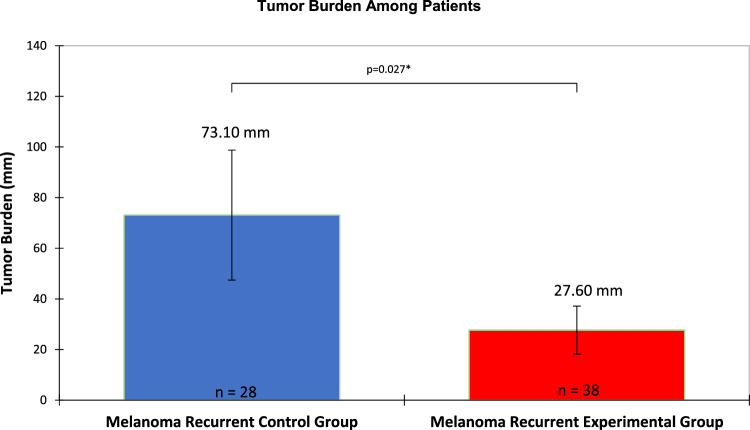
Bar diagram representing the average tumor burden (measured in mm) between the control group and experimental group

**Fig. 3:**
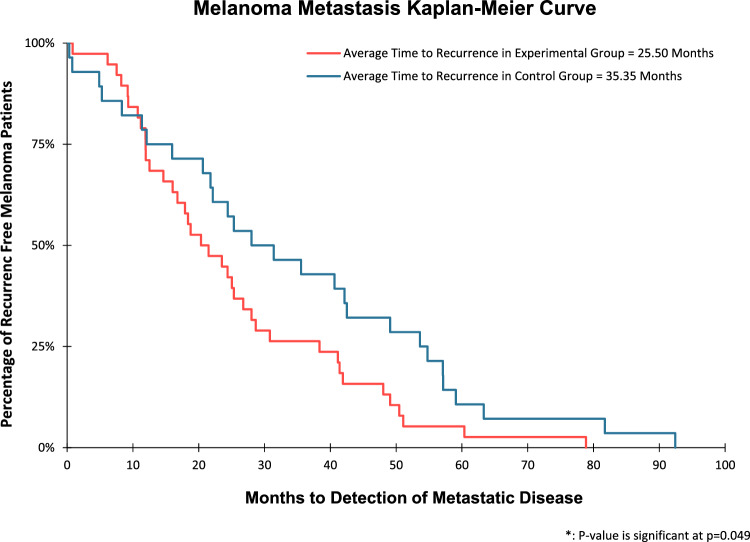
Kaplan–Meier curve illustrating percentage of recurrence free patients over time

The average tumor burden among recurrent melanoma patients in the experimental group was 27.60 mm compared to 73.10 mm in recurrent melanoma patients from the control group (Fig. 2), which was statistically significant with a p-value of 0.027. Kaplan–Meier analysis (Fig. 3) also showed that the time difference between the detection of melanoma recurrence in the experimental group and control group was significant; on average, melanoma recurrence was detected at 25.50 months in the experimental group versus 35.35 months for the control group (p-value of 0.049; t-test p-value of 0.004).

Among patients with recurrent melanoma, the experimental cohort had a greater average Breslow depth (3.72 mm vs. 3.31 mm), older age (65.75 years vs. 59.2 years) at primary diagnosis, and a higher proportion of clinical stage 2 patients (89.5% vs. 71.4%) compared to the control group. None of these findings met statistical significance (Table 1). There were also no significant differences regarding gender, initial tumor site, and distant recurrences to brain, lymph nodes, and total visceral recurrences. Lungs were the most common distant metastatic site, present in 65.2% of all melanoma recurrent patients, followed by lymph nodes with 31.8%.

**Table 1:** Demographic summary table of control and experimental groups

All patients	Control group	Experimental group	Total	P-value
	(N = 327)	(N = 307)	(N = 634)	
Melanoma Recurrence	14.1% (46/327)	20.5% (63/307)	17.1% (109/634)	0.031^a^

Recurrent melanoma patients	Control group patients (N = 28)	Experimental group patients (N = 38)	Total (N = 66)	P-value
Average breslow	3.31 mm	3.72 mm	3.55 mm	0.171^b^
Tumor staging at primary diagnosis				0.060^a^
Clinical Stage I				
T1a	1	0	1	
T1b	2	0	2	
T2a	5	4	9	
Total Stage I	28.6% (8/28)	10.5% (4/38)	18.2% (12/66)	
Clinical Stage ≥ II				
T2b	2	4	6	
T3a	7	6	13	
T3b	6	10	16	
T4a	1	3	4	
T4b	4	11	15	
Total Stage ≥ II	71.4% (20/28)	89.5% (34/38)	81.8% (54/66)	
Sex				
Male	17	19	36	0.388^a^
Female	11	19	30	
Age at primary diagnosis				
Male mean	58.75	66.81	63.01	0.090^b^
Male range	27–85	49–89	27–89	
Female mean	60.05	64.69	62.99	0.880^b^
Female range	41–82	30–81	30–82	
Both sexes mean	59.20	65.75	63	0.181^b^
Both sexes range	27–85	41–89	27–89	
Sites of recurrence^c^				0.133^a^
Lungs	57.1% (16/28)	71.1% (27/38)	65.2% (43/66)	0.241^a^
Liver	17.9% (5/28)	2.6% (1/38)	9.1% (6/66)	0.033^a^
Brain	10.7% (3/28)	15.8% (6/38)	13.6% (9/66)	0.553^a^
Lymph nodes	25.0% (7/28)	36.8% (14/38)	31.8% (21/66)	0.451^a^
Bone	7.1% (2/28)	0.0% (0/38)	3.0% (2/66)	0.094^a^
Intestine	0.0% (0/28)	5.3% (2/38)	3.0% (2/66)	0.218^a^
Patients with visceral sites of recurrence	85.7% (24/28)	92.1% (35/38)	89.4% 59/66	0.405^a^
Site of initial tumor				0.781^a^
Head/neck	28.6% (8/28)	28.9% (11/38)	28.8% (19/66)	0.973^a^
Upper extremity	25.0% (7/28)	28.9% (11/38)	27.3% (18/66)	0.722^a^
Trunk	28.6% (8/28)	18.4% (7/38)	22.7% (15/66)	0.331^a^
Lower extremity	17.9% (5/28)	23.7% (9/38)	21.2% (14/66)	< 0.001^a^
Immunotherapy^d^				
Number of patients	71.4% (20/28)	81.6% (31/38)	77.3% (51/66)	0.331^a^
Patient status				
Alive patients	50.0% (14/28)	76.3% (29/38)	65.2% (43/66)	0.027^a^
Deceased patients	50.0% (14/28)	23.7% (9/38)	34.8% (23/66)	

^a^Chi-Square p-value

^b^Kruskal-Wallis p-value

^c^Calculation analyzed total number of recurrences; some patients had multiple sites of recurrence upon discovery

^d^Patients who did not start immunotherapy when offered were excluded

The percentage of patients who started immunotherapy was 81.6% (31/38) among recurrent melanoma patients in the experimental group and 71.4% (20/28) among recurrent melanoma patients in the control group. At the time of the last follow-up, 76.3% (29/38) of the patients with melanoma recurrence in the experimental group were alive with an average follow-up time of 45.63 months, compared to 50.0% (14/28) of recurrent melanoma patients in the control group with an average follow-up time of 63.32 months. The difference in overall survival was statistically significant, with a chi-square p-value of 0.027.

## Discussion

Historically, early detection of metastatic disease was considered unjustified, given the lack of effective treatment options prior to the recent advances in of systemic therapies [13]. However, recent studies suggest a survival benefit when metastatic melanoma is treated at a lower tumor burden [4–8]. The COMBI-d and COMBI-v trials showed a significant improvement in OS and PFS in patients treated with dabrafenib plus trametinib (MEK inhibitor) when patients started at less than 3 tumor sites (n = 282) versus more than or equal to 3 tumor sites (n = 269) [25]. Likewise, in studies of patients treated with the anti-PD-1 antibody, pembrolizumab, OS decreased as the number of metastatic lesions increased, and patients with a longer PFS generally had a lower tumor burden [6, 26]

In a retrospective medical imaging review of 10 patients with metastatic melanoma treated with dabrafenib, the mortality hazard tripled for every 1 cc increase in tumor volume (p = 0.047, HR 2.81, CI 1.06 –6.19), and patients with tumor volumes above the median of 111.5 cc also had a statistically significant shorter OS than patients with smaller tumor volumes (6 months vs. 56 months, p-value = 0.019) [27].

In contrast to using tumor volumes or sites, our study used cumulative tumor size to measure tumor burden. We calculated the clinical tumor burden as the sum of the largest unidimensional lengths of all metastatic foci. Specifically, we compared the tumor burden at the time of the first evidence of melanoma recurrence in a cohort of patients with stage 1 or 2 disease and with GEP Class 2A/B accompanied by routine imaging with a cohort of patients with stage 1 or 2 disease melanoma without GEP testing or routine imaging. Despite having a higher mean Breslow and a higher proportion of patients with clinical stage II disease or greater, the experimental group had a significantly lower tumor burden detected at the first recurrence (27.60 mm verse 73.10 mm).

As a secondary endpoint we assessed overall survival at the time of last follow up. In accordance with the higher rate of recurrence in the experimental group, more patients with recurrent melanoma in the experimental group were started on immunotherapy. Importantly, patients in the experimental group had statistically significant better overall survival (76.3% versus 50.0%). It is important to note that there was a difference in average follow-up times between the control and experimental groups, with an average follow-up of 63.32-months and 45.63 months, respectively. Given that the follow-up time was longer in the control group, this may have led to more deaths reported. Therefore, we analyzed both melanoma recurrent experimental and control group patients at the 45.63-month mark, which showed a similar trend in results, though not significant. Specifically, at a maximum follow-up of 45.63 months, 86.80% of the melanoma recurrent experimental patients were alive, and 75.00% of the melanoma recurrent control group patients were alive. A trend towards improved overall survival in patients with recurrent melanoma of the experimental group supports previous studies suggesting that response to newer systemic therapies may be better when tumor burden is lower [4–7]. Hence, in the current era of novel targeted and immunotherapy there may be a need for greater emphasis on detecting metastatic disease earlier.

Past studies evaluating the impact of imaging studies on early detection of recurrent diseases have had mixed results. This may be related to large sample sizes that included patients with minimal risk of metastasis [28]. As expected, when implementing an interventional strategy in cohorts of patients with minimal risk of metastasis, the odds of finding a statistically significant benefit are low. [29, 30]. However, more are recent studies suggest that in higher risk patients, routine imaging can identify early visceral or lymphatic melanoma metastasis in clinically asymptomatic patients [13, 31]. In our study, we found that surveillance imaging detected melanoma recurrence 9.84 months earlier (25.50 months vs. 35.35 months) in patients who had routine imaging schedules compared to those who did not. This included visceral (92.1%), nodal (36.8%) and CNS (15.8%) recurrences. Therefore, surveillance imaging can detect melanoma recurrence in high-risk patients at an earlier time frame with a lower overall tumor burden.

Compared to routine clinical exams, imaging studies are more costly and should be used strategically according to the patient's risk of recurrence [9]. In fact, NCCN guidelines recommend that follow-up of patients be based on their level of risk of relapse (13). The 31-GEP test is a tool for identifying the risk of melanoma relapse, which has been particularly shown to identify a high risk of recurrence in patients with clinical stage 1 or 2 AJCC disease [32]. In a study of 259 patients with negative SLNBx, 70% of patients with high risk of recurrence (Class 2) 31-GEP results experienced metastasis [32]. In our study of patients with stage 1 and 2 disease, significantly more patients with high score 31-GEP results experienced melanoma recurrence compared to those without 31-GEP testing (20.5% vs. 14.1%). Therefore, the 31-GEP tool may offer one strategy of identifying high risk stage 1 and 2 patients who ultimately account for a significant proportion of melanoma related deaths.

The limitations of our study included the retrospective nature of this study, with a limited sample size of patients with recurrent melanoma. In addition, there were less than uniform imaging protocols among all three sites, including the type of imaging study recommended. There was a difference in the durations of the surveillance intervals, which ranged from 6 to 12 months. Furthermore, among all three sites and both groups, there was also a difference in the patient follow-up lengths for patients with recurrence.

In summary, the 31-GEP test identifies patients who are at a higher risk of developing metastases, and when combined with routine imaging studies, patients with visceral or lymphatic metastases can be identified and offered systemic and immunotherapy treatment in an earlier time frame with a lower tumor burden, which can lead to improved patient outcomes.

## Data Availability

The data generated in this study are not publicly available but deidentified data are available upon reasonable request from the corresponding author.
