# Correction to: Routine imaging guided by a 31‑gene expression profile assay results in earlier detection of melanoma with decreased metastatic tumor burden compared to patients without surveillance imaging studies

**DOI:** 10.1007/s00403-023-02622-5

**Published:** 2023-04-12

**Authors:** Soneet Dhillon, Daniela Duarte-Bateman, Graham Fowler, Michael Norman Eun Hagstrom, Nathaniel Lampley, Shantel Olivares, Mónica Stella Fumero-Velázquez, Kathryn Vu, Jeffrey D. Wayne, Brian R. Gastman, John Vetto, Pedram Gerami

**Affiliations:** 1 Department of Dermatology, Feinberg School of Medicine, Northwestern University, 676 N. St. Clair Street, Suite 1765, Chicago, IL 60611 USA; 2 Department of Plastic Surgery, Cleveland Clinic Lerner Research Institute, Cleveland, OH USA; 3 Division of Surgical Oncology, Knight Cancer Institute, Oregon Health and Science University, Beaverton, USA; 4 Division of Surgical Oncology, Northwestern University, Chicago, IL USA; 5 Robert H. Lurie Cancer Center, Feinberg School of Medicine, Northwestern University, Chicago, IL USA

**Correction to: Archives of Dermatological Research** 10.1007/s00403-023-02613-6

In the sentence beginning ‘However, melanoma recurrence was…’ of the Abstract section in this article, the text ‘in the experimental group at a lower overall tumor burden (73.10 mm versus 27.60 mm)’ should have read ‘in the experimental group at a lower overall tumor burden (27.60 mm versus 73.10 mm)’.

