# Correction: Routine imaging guided by a 31‑gene expression profile assay results in earlier detection of melanoma with decreased metastatic tumor burden compared to patients without surveillance imaging studies

**DOI:** 10.1007/s00403-023-02733-z

**Published:** 2023-11-25

**Authors:** Soneet Dhillon, Daniela Duarte-Bateman, Graham Fowler, Michael Norman Eun Hagstrom, Nathaniel Lampley, Shantel Olivares, Mónica Stella Fumero-Velázquez, Kathryn Vu, Jeffery D. Wayne, Brian R. Gastman, John Vetto, Pedram Gerami

**Affiliations:** 1 Department of Dermatology, Feinberg School of Medicine, Northwestern University, 676 N. St. Clair Street, Suite 1765, Chicago, IL 60611 USA; 2 Department of Plastic Surgery, Cleveland Clinic Lerner Research Institute, Cleveland, OH USA; 3 Division of Surgical Oncology, Knight Cancer Institute, Oregon Health and Science University, Beaverton, USA; 4 Division of Surgical Oncology, Northwestern University, Chicago, IL USA; 5 Robert H. Lurie Cancer Center, Feinberg School of Medicine, Northwestern University, Chicago, IL USA

**Correction: Archives of Dermatological Research (2023) 315:2295–2302** 10.1007/s00403-023-02613-6

The article Routine imaging guided by a 31‑gene expression profile assay results in earlier detection of melanoma with decreased metastatic tumor burden compared to patients without surveillance imaging studies, written by Soneet Dhillon, Daniela Duarte‑Bateman, Graham Fowler, Michael Norman Eun Hagstrom, Nathaniel Lampley, Shantel Olivares, Mónica Stella Fumero‑Velazquez, Kathryn Vu, Jeffrey D. Wayne, Brian R. Gastman, John Vetto and Pedram Gerami, was originally published Online First without Open Access. After publication in volume 315, issue 8, pages 2295–2302 the author decided to opt for Open Choice and to make the article an Open Access publication. Therefore, the copyright of the article has been changed to © The Author(s) 2023 and the article is forthwith distributed under the terms of the Creative Commons Attribution 4.0 International License, which permits use, sharing, adaptation, distribution and reproduction in any medium or format, as long as you give appropriate credit to the original author(s) and the source, provide a link to the Creative Commons licence, and indicate if changes were made. The images or other third party material in this article are included in the article’s Creative Commons licence, unless indicated otherwise in a credit line to the material. If material is not included in the article’s Creative Commons licence and your intended use is not permitted by statutory regulation or exceeds the permitted use, you will need to obtain permission directly from the copyright holder. To view a copy of this licence, visit http://creativecommons.org/licenses/by/4.0.

